# Impact of growth implants and low-level tannin supplementation on enteric emissions and nitrogen excretion in grazing steers

**DOI:** 10.1093/tas/txae115

**Published:** 2024-07-31

**Authors:** Edward J Raynor, Mesa Kutz, Logan R Thompson, Pedro H V Carvalho, Sara E Place, Kimberly R Stackhouse-Lawson

**Affiliations:** AgNext, Department of Animal Sciences, Colorado State University, Fort Collins, CO 80523, USA; AgNext, Department of Animal Sciences, Colorado State University, Fort Collins, CO 80523, USA; Department of Animal Sciences and Industry, Kansas State University, Manhattan KS 66506, USA; AgNext, Department of Animal Sciences, Colorado State University, Fort Collins, CO 80523, USA; AgNext, Department of Animal Sciences, Colorado State University, Fort Collins, CO 80523, USA; AgNext, Department of Animal Sciences, Colorado State University, Fort Collins, CO 80523, USA

**Keywords:** enteric methane emissions, emissions intensity, grazing improved pasture, greenhouse gas mitigation options, mitigation strategies, quebracho and chestnut tannin extract

## Abstract

The primary objective of this experiment was to evaluate the effects of a growth-hormone implant (Revalor-G, Merck Animal Health., Rahway, NJ, USA) and tannin supplementation (Silvafeed BX, Silva Team, San Michele Mondovi CN, Italy) on enteric methane (CH_4_) emissions and estimated nitrogen (N) excretion in grazing steers. Steers (*n* = 20; initial body weight [IBW] = 343 ± 14 kg) were acclimated to use a portable automated head-chamber system (AHCS) to measure CH_4_ and a SmartFeed Pro automated feeder for dietary supplementation (C-Lock Inc., Rapid City, SD, USA). After the training period, steers were randomly assigned to a 2 × 2 factorial arrangements of treatments, with 2 levels of growth-hormone implants, no-implant (NO-IMP) or implanted (IMP), and 2 levels of tannin supplementation, no tannin supplementation (NO-TAN) or tannin supplementation (TAN). This created 4 treatment groups: (1) NO-TAN and NO-IMP, (2) TAN and NO-IMP, (3) IMP and NO-TAN, and (4) TAN and IMP. Tannin was offered daily at 0.30% dry matter intake (DMI) through 0.5 kg/hd/d sweetfeed supplement (Sweetfeed Mix, AgFinity., Eaton, CO, USA) with a targeted tannin intake at 48 g/hd/d. No (*P* ≥ 0.05) implant × tannin interaction was detected for any dependent variable, so only the main effects of implant (NO-IMP vs. IMP) and tannin supplementation (NO-TAN vs. TAN) are discussed. Implant status did not affect (*P* ≥ 0.56) final body weight (FBW) or average daily gain (ADG) during the 90 d grazing period. There was no effect (*P* ≥ 0.15) of growth implant on CH_4_ production or emission intensity (EI; g CH_4_/kg gain). Additionally, IMP steers tended (*P* ≤ 0.08) to have less CH_4_ yield (MY; g CH_4_/g DMI) and higher blood urea nitrogen (BUN) than NO-IMP steers. Tannin supplementation did not impact (*P* ≥ 0.26) FBW or ADG. However, NO-TAN steers tended (*P* = 0.06) to have a greater total DMI than steers supplemented with tannin. No effect (*P* ≥ 0.22) of tannin supplementation was observed for CH_4_ production and EI. Nitrogen utilization as measured through BUN, urine N, fecal N, or fecal P was similar (*P* ≥ 0.12) between TAN and NO-TAN animals. The findings indicate that low-level dietary supplementation to reduce enteric emissions is difficult in grazing systems due to inconsistent animal intake and that growth implants could be used as a strategy to improve growth performance and reduce EI of steers grazing improved pasture.

## Teaser

Enteric methane emissions per unit of average daily gain were reduced 7% by providing grazing steers with a growth-promoting implant, while supplementing steers with a low level of commercially available source of tannins did not reduce emissions.

## Introduction

A growing concern exists regarding the contribution of livestock production to anthropogenic greenhouse gas (GHG) emissions, primarily driven by enteric methane (CH_4_) and manure nitrous oxide (N_2_O). In 2021, the beef production sector was directly responsible for 2.4% of total US emissions, 19.4% of CH_4_, and 2.1% of N_2_O emissions from the combined sources of manure management and enteric fermentation (EPA 2023). In the most recent life cycle assessment for U.S. beef production, the U.S., grazing cattle—the cow-calf and stocker sectors combined—contribute 89% of CH_4_ emissions and 83% of N_2_O emissions from the beef sector and represent an opportunity for mitigation (Rotz et al., 2019). These grazing animals also supply 20% of global beef production and support most of the US breeding herd (Rotz et al., 2019). The ability of grazing cattle to convert complex carbohydrates with high fiber content on untillable land into useable end products such as meat is a unique advantage, and it is crucial to the economic, social, and environmental sustainability of the beef supply chain and maintaining food security (Gerber et al., 2015; Carvalho et al., 2018; Thompson and Rowntree, 2020). Therefore, it is necessary to develop strategies that can effectively mitigate GHG emissions without compromising animal performance.

Many potential mitigation strategies have been reviewed (Berndt and Tomkins, 2013; Gerber et al., 2013; Hristov et al., 2013; Thompson and Rowntree, 2020), including the use of supplementation with plant secondary compounds (i.e., condensed and hydrolysable tannins) and growth-promoting technologies (i.e., growth hormone implants). However, validation of such strategies for reducing enteric emissions has been mostly limited to confined settings (Beauchemin et al., 2007; Schilling et al., 2024) or in vitro studies (Tan et al., 2011) without attention to efficacy for use with grazing beef cattle. Further, stacking of multiple technologies to reduce GHG emissions from ruminants has also been evaluated in the literature (Rotz, 2020; Bilotto et al., 2023) yet exploration of this approach is still in its infancy (Makate et al., 2019; Harrison et al. 2021), with little focus on implementation in grazing systems.

Although literature on tannins reducing CH_4_ production is variable, many studies have reported that the inclusion of tannins in the diet shows promise for decreasing CH_4_ emissions in ruminants (Min et al., 2003; Carulla et al., 2005; Bhatta et al., 2009). Tannins are a diverse group of plant secondary compounds that interact with ruminal nitrogen (N) and fiber fermentation (Min et al., 2003; Carulla et al., 2005) and include both condensed and hydrolysable tannins, which have the ability to reduce enteric CH_4_ emissions due to their ability to bind proteins and carbohydrates within the rumen (McSweeney et al., 2001; Min et al., 2003). This binding action inhibits microbial attachment, reducing ruminal fiber fermentation while increasing the availability of bypass protein, which could improve protein digestion and decrease N excretion (Min et al., 2003). Marshall et al. (2022) demonstrated that providing a blended condensed and hydrolysable tannin supplement reduced the abundance of methanogenic archaea *Methanobacteriaceae* by 16% and *Methanosarcinaceae* by 30% compared to non-supplemented beef cattle. This suggests that supplementing beef cattle with tannin may reduce GHG emissions and environmental impacts associated with N excretion due to this change in microbiome populations.

Standard beef cattle management practices have also been evaluated for their GHG mitigation potential (Stackhouse et al., 2012; Hristov et al., 2013; Thompson and Rowntree, 2020). Anabolic growth implants are one common growth-promoting technology used by producers. For instance, more than 75% of stocker cattle are implanted due to the benefit of increased animal performance (Kuhl, 1996; Selk et al., 2006; Asem-Hiablie et al., 2015; Reinhardt and Thomson, 2016; Beck et al., 2022). Revalor-G (REV-G, Merck Animal Health, Rahway, NJ, USA) is a medium-potency growth implant that contains 40 mg of trenbolone acetate and 8 mg of estradiol in a slow-release delivery system that has been shown to improve ADG within 32 d post-implementation (Kuhl and Blasi., 1998). As ADG increases, emissions intensity (EI; g CH_4_/kg gain) can decrease (McAuliffe et al., 2018). Therefore, if the growth implant increases ADG, there is potential for a decrease in emissions intensity ([EI;g CH_4_/kg BW gain]; Stackhouse et al., 2012), yet limited empirical work has been done examining CH_4_ production in conjunction with implant use, representing a significant knowledge gap in the literature. In the current study, we hypothesize that utilizing a growth hormone in grazing steers will result in reductions in CH_4_ emissions per unit of body weight gain, and supplemental tannin will have an additive effect on this reduction. Additionally, N excretion will be shifted and result in less urinary N and fecal N excretion. Considering the large contribution of grazing cattle to enteric CH_4_ emissions, the primary objective of this experiment was to understand how implanting with REV-G and supplementing with a blend of chestnut and quebracho tannins (Silvafeed BX, Silva Team, San Michele Mondovi CN, Italy) impacts enteric CH_4_ and N utilization in grazing steers.

## Materials and Methods

The experimental procedures were approved by the Institutional Animal Care and Use Committee at Colorado State University (CSU; IACUC #3356). All cattle under the care of this study were maintained and managed following all guidelines outlined in the protocol and this document and with the utmost care and humane handling.

### Experimental Location

**Figure 1.**
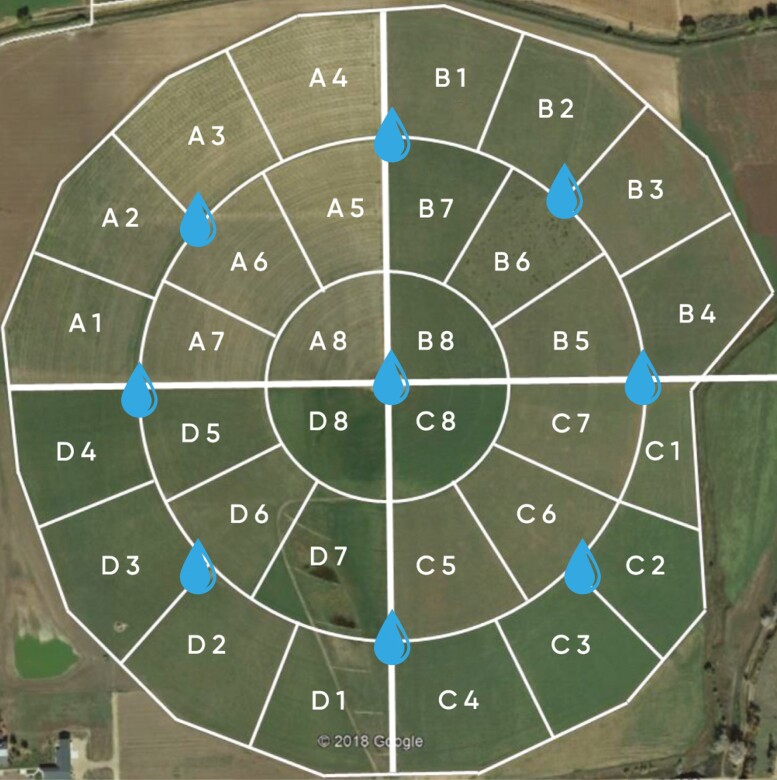
Sections within the center pivot-irrigated pasture at the Colorado State University (CSU) Agricultural Research, Development, and Education Center (ARDEC) in Fort Collins, Colorado (Modified from Shawver et al. 2021). *F = sections of the pivot-irrigated pasture where forage was collected. White lines represent section electric fencing perimeter, while water drops represent location of section waterers.

The experiment was conducted between June 19 and September 18, 2022 (90 d) on an 82-ha center pivot-irrigated pasture located at the Colorado State University (CSU) Agriculture, Research, Development, and Education Center (ARDEC; Figure 1, 40° 65ʹN, 105° 00W, 1557.528 m asl). The local climate is a mid-latitude dry, cold, semiarid steppe (Kottek et al., 2006), with average low and high temperatures of 1.0 and 16.8 °C, average annual rainfall of 350 mm for 1992 to 2022 with the average for June to September of 320 mm (CoAgMET 2023). During the experiment, there was a total precipitation of 116 mm, and the mean air temperature was 21.7 °C. The center pivot-irrigated pasture contained 31 grazing sections ranging from 2.19 to 3.08 ha. The sections were predominantly cool-season perennial grasses, including orchard grass (*Dactylis glomerata*), tall fescue (*Festuca arundinacea*), meadow fescue (*Festuca pratensis*), perennial ryegrass (*Lolium perenne*), meadow brome (*Bromus biebersteinnii*), smooth brome (*Bromis inermis*), and alfalfa (*Medicago sativa*) (Shawver et al., 2021). Steers were rotated on 2 to 4 d intervals depending on forage availability in a graze and follows grazing management plan with the resident cow-herd at a stocking density of 7.33 animal units/ha. Therefore, steers in the current study were managed to follow the resident cow herd as they moved from section to section in the center pivot-irrigated pasture. Moves were dictated by forage regrowth to maintain adequate dry matter intake (DMI). Animals were offered ad libitum access to drinking water and commercially available free-choice mineral block. Further description of the study site and pasture establishment is described by Shawver et al. (2021).

### Measurement of Forage Availability and Chemical Analysis

Forage samples were taken every 2 wk for the duration of the study using the quadrat method (Fig. 1; Thompson et al., 2021). Prior to herd rotation, pregrazed samples were collected by randomly placing six 0.25-m^2^ quadrats and clipping them to a 5-cm stubble height in each experimental section. All clippings were weighed wet, dried in a 65 °C oven for 3 d, and weighed again to calculate dry matter (DM) content. Dry matter content was then used to calculate forage availability. Samples were then ground to pass through a 1-mm screen (Thomas A. Wiley Laboratory Mill, Swedesboro, NJ, USA) and composited by weight prior to forage Near-Infrared spectroscopy (NIRS) analysis at a commercial laboratory (Dairy One; Ithaca, NY, USA; Table 1). Composite samples were then analyzed for crude protein (CP), neutral detergent fiber (aNDF), acid detergent fiber (ADF), lignin, total digestible nutrients (TDN), net energy for maintenance (NEm), net energy for growth (NEg), and neutral detergent fiber digestibility (NDFD).

**Table 1.** Nutritive content (% DM basis) of forage grazed by growing steers during the June to September 2022 grazing season at Colorado State University Agricultural Research, Development and Education Center (ARDEC), Fort Collins, CO, USA

	Day^1^^,^^2^						
Item	0	14	28	42	56	70	84
DM, % as fed	36.8	34.6	27.8	38.9	31.2	39.9	31.7
CP, % DM	16.3	18.0	20.0	8.4	25.8	12.0	12.5
ADF, % DM	29.1	31.7	35.9	44.5	29.1	39.8	35.8
NDF, % DM	50.8	52.7	49.7	65.1	46.1	61.0	55.7
TDN, % DM	70	69	68	56	68	65	69
Lignin, % DM	3.2	3.6	4.8	6.4	5.3	4.4	3.1
NEm, Mcal/kg	0.70	0.68	0.67	0.49	0.68	0.62	0.68
NEg, Mcal/kg	0.43	0.42	0.41	0.24	0.41	0.36	0.41
NDFD, % of NDF	79	77	68	51	82	62	74

^1^Forage samples collected every 2 wk (June 19 to September 18, 2022).

^2^On average, forage availability was 1,480 kg DM/ha.

### Animals, Treatments, and Design

Thirty crossbred Angus steers (BW = 358 ± 43 kg), all originating from the John E. Rouse-CSU Beef Improvement Center near Saratoga, WY, USA, were enrolled in the grazing experiment. Prior to emissions measurements on the pivot-irrigated improved pasture, steers were acclimated to a portable, automated head-chamber system (AHCS; Greenfeed, C-Lock Inc., Rapid City, SD, USA), and portable SmartFeed Pro self-feeder (C-Lock Inc., Rapid City, SD, USA) for 3 wk. Twenty steers (BW = 343 ± 14 kg) were selected for the experiment based on the acclimation rate to both the AHCS and Smartfeed Pro. After the training period, steers were randomly assigned to a 2 × 2 factorial arrangements of treatments; tannin supplemented (Silvafeed BX, Silva Team, San Michele Mondovi CN, Italy) at 0.30% DM intake/day (TAN) or no tannin supplementation (NO-TAN); and a growth hormone implant program (IMP) with 40 mg of trenbolone acetate and 8 mg of estradiol (Revalor-G, Merck Animal Health, Rahway, NJ, USA) or no growth hormone implant (NO-IMP). Body weights (BW) were collected prior to study initiation and every 30-d for the duration of the experiment using a hydraulic squeeze chute (Silencer, Moly Manufacturing, Lorraine, KS, USA) and weigh scale (Tru-Test Scale, Tru-Test Inc., Mineral Wells, TX, USA) at the ARDEC working facility. An additional weight measurement occurred halfway through the study at day 45, which ended the early period (days 0 to 45) and began the later period (days 46 to 90) of the study. A switch from an earlier model AHCS to a later model AHCS unit occurred on day 45.Average daily gain was determined via the slope coefficient of a linear regression model as a function of gain and day.

At the beginning of the study, steers were injected with a 7-way clostridial vaccine (Vision 7; Merck Animal Health, Rahway, NJ, USA), vaccinated with a modified live, 5-way vaccine for infectious bovine rhinotracheitis, bovine respiratory parainfluenza-3, bovine virus diarrhea types 1 and 2, *Mannheimia heamolytica* and *Pasteurella multocida* (Vista Once; Merck Animal Health, Rahway, NJ, USA), dewormed (Safeguard; Merck Animal Health, Rahway, NJ, USA), treated with a pour-on topical insecticide (Cyonara Plus; Control Solutions Inc., Pasadena, TX, USA) and then randomly assigned to one of the 4 treatment levels: (1) NO-TAN and NO-IMP, (2) TAN and NO-IMP, (3) NO-TAN and IMP, and (4) TAN and IMP.

The tannin supplement was fed using a sweetfeed mix at 0.5 kg/hd/d using the SmartFeed Pro self-feeder (Table 2; Sweet Mix, AgFinity, Eaton, CO, USA); treatment groups without tannin received the same sweetfeed ration at 0.5 kg/hd/d without tannin supplementation. The tannin was targeted at 0.3% DMI and calculated at 48 g/hd/d for the duration of the study.

**Table 2.** Ingredient and nutrient content of alfalfa pellet and supplement

Item	Alfalfa pellet^1^	Supplement^2^
Nutritive value, %DM		
CP	21.4	11.17
ADF	34.4	4.69
Lignin	6.7	1.27
aNDF	42.6	9.01
NEm	0.63	0.94
NEg	0.37	0.64
NDFD, % of NDF	48	54
DM, % as offered	91.4	94.4

^1^Supplier (AgFinity; Eaton, CO, USA) did not supply an ingredient list; alfalfa pellet was used as AHCS bait.

^2^Supplier (AgFinity; Eaton, CO, USA) did not supply ingredient list; sweetfeed mix was used as base feed supplement mixed with tannin supplement and was fed out of the Smartfeed Pro.

### Equipment Acclimation Period

The AHCS system allows for measurement of CH_4_ and CO_2_ emission and O_2_ consumption. Acclimation for the AHCS was done according to Gunter and Beck (2018). During the acclimation period, the AHCS was initially introduced with no panels or windbreaks around the machine. Panels were then added at a wide distance and slowly placed closer together until only one animal could fit in the alley leading up to the AHCS animal inlet. The AHCS uses bait to attract and occupy the animal while gas flux measurements are taken. In the current study, the bait was alfalfa pellets. During the acclimation period, the animals were kept in a 0.09-ha feedlot pen and offered a forage-based diet. The SmartFeed Pro allows cattle to be supplemented on pasture and has a specially designed door that allows for controlled individual animal intake. At the beginning of acclimation, the SmartFeed Pro had the automatic head gates locked open to allow ad libitum access for each animal. Two weeks into acclimation, the automatic head gates on the automated feeder were raised to allow acclimation to the feeder’s doors. After 30 d, all steers were moved onto the pivot-irrigated pasture to allow for acclimation to the pasture and electric fence for 10 d. While on pasture, animals were assigned access to one of 2 feeders of the SmartFeed Pro. After acclimation, 20 steers were selected based on the acclimation rate to both the AHCS and SmartFeed Pro to be used in the experiment.

### Measurement of Forage Intake

Forage intake was estimated using an intake prediction equation.

Nutrient requirements of beef cattle equation for all-forage diets (NRC, 2000, p. 94, Eq: CP__ADF)


$$\begin{array}{lll} \frac{DMI\;kg}{kg\;SBW^{0.75}}=\; & 0.002774\;\times \;forage\;CP\%-0.000864\; & \times \;forage\;ADF\%\;+0.09826 \end{array}$$

Percentage of CP and ADF in the forage are expressed on a dry matter basis. Kilograms of SBW^0.75^ (metabolic BW based on shrunk BW) was converted to DMI (kg/d). Dry matter intake in kilogram per day was then calculated.

### Measurement of Emissions

Due to operating challenges with the AHCS system during our acclimation period, the AHCS started collecting data on day 9 (June 29, 2022). The AHCS recorded individual CH_4_ and CO_2_ emissions and O_2_ consumption each time an animal visited using the cattle’s radio frequency identification (RFID) during data collection. A more advanced AHCS replaced the original AHCS halfway through the study, day 45, that also collected H_2_ emission. To measure CH_4_ and CO_2_ concentrations, the initial AHCS employed a non-dispersive-infrared sensor, while the more advanced AHCS used a tunable diode laser. Carbon dioxide recoveries were conducted on both systems to ensure no difference would occur when switching AHCS and both had recoveries of 100 ± 5%. Animals were allowed a maximum of6 visits each day, with a maximum of 6 drops each visit (Gunter and Bradford, 2017; Gunter and Beck, 2018). On average, a single drop of the alfalfa pellet bait weighed 34 g (mean ± SD: 34 ± 1.3 g per drop) and there were 30 s intervals between drops. This was set to encourage the animal to remain in the AHCS for a minimum of 3 min (Velazco et al., 2016). All visits that recorded less than 3 min and greater than 8 min were removed from analysis. The minimum time between visits was 4 h to ensure distribution over 24 h periods to capture diurnal variation in CH_4_ production (Della Rosa et al., 2021). The alfalfa pellet bait feed (9.5-mm diameter pellets, approximately 34 g/dispense; AgFinity; Eaton, CO, USA) were sampled monthly and analyzed for nutritive value using NIRS analysis by a commercial laboratory (Dairy One; Ithaca, NY, USA; Table 2). During each visit, the animal would enter a narrow panel system to ensure that only one animal was visiting the machine. Once the animal inserted its head into the AHCS, the system would scan and register the animal’s RFID and begin dropping the pelleted bait feed. The process of CH_4_ and CO_2_ measurements by the AHCS used are further described by Hristrov et al. (2015). The AHCS was auto-calibrated weekly. CO_2_ recoveries were completed before, during, and after the study with recoveries of 100 ± 5%. For analysis, individuals with ≥10 good visits (Thompson et al., 2021) were selected for final analysis within the period (2 animals had less than 10 visits and were removed from analysis).

CH_4_ emissions were also estimated using two different prediction equations to compare measured results from the current experiment to estimated results from prediction equations. The IPCC Tier 2 (2019) equation for animals consuming a >75% forage diet used DMI and an estimated CH_4_ yield value as factors to calculate the predicted CH_4_. Dry matter intake was calculated based on the NRC (2000) predicted intake equation previously described. The IPCC Tier 2 (2019) equation was then converted to g of CH_4_ per animal per day:

IPCC Tier 2 (2019) equation for animals consuming a > 75% forage diet:


$$\begin{array}{l} EF=DMI\times \;\;\;\left(\frac{MY}{1,000}\right)\times 365 \end{array}$$ (1)

where emission factor (EF) represents kg CH_4_ per head per year, DMI represents kg DMI per day, 365 represents the days in a year, and 1,000 represents the conversion from g of CH_4_ to kg of CH_4_.

Thompson et al. (2019) used a regression model to predict CH_4_ production in which stocker cattle were grazing wheat (*Triticum aestivum*). Dry matter intake of forage was estimated using the NRC (2000) equation.

Predictive methane equation (Thompson et al., 2019):


$$\begin{array}{l} CH_{4}=98.33+0.17\times IBW+5.22\times FI-11.31\times SEX \end{array}$$ (2)

where IBW represents the initial body weight, FI represents DMI of the forage, and SEX is 1 for heifers and 0 for steers.

### Blood, Urine, and Fecal Analyses

Blood was drawn from the jugular vein on days 0, 45, and 90 after one 12-h fasting period from steers in a squeeze chute. Blood was collected in EDTA tubes (BD Vacutainer EDTA blood tube; Fisher Scientific, Pittsburgh, PA, USA) and centrifuged for 10 min at 210 × *g* at 4 °C. Serum was removed and stored at −80 °C. At the conclusion of the study, all serum samples were sent to the CSU Diagnostic Laboratory (Fort Collins, CO, USA) for blood urea nitrogen (BUN) analysis.

Urine was collected via manual stimulation from steers in a squeeze chute on days 0, 45, and 90. Once collected, each urine sample was placed in 50 mL tube with 10 mL of HCl and frozen at 4 °C. After the study was completed, all samples were sent to Ward Laboratories, Kearney, NE for urine N analysis and CSU Veterinary Clinical Pathology Laboratory (Fort Collins, CO, USA) for urine creatinine analysis.

Fecal samples were collected via rectal grab on days 0, 45, and 90 while steers were in the squeeze chute. Samples were placed in quart-sized Ziploc bags and transported to CSU ARDEC laboratory facilities. Samples were weighed wet, dried in a 65 °C oven for 3 d, and weighed again to calculate DM content. Dried fecal samples were ground through a 1-mm screen (Thomas A. Wiley Laboratory Mill, Swedesboro, NJ, USA) and placed in Whirl PAK bags to prevent sample contamination until further analysis. Samples were sent to Ward Laboratories (Kearney, NE, USA) for fecal N and P analysis.

### Statistical Analysis

One tannin-supplemented steer gained significantly less (approximately <50%) than all other animals during the 90-d of the study and was excluded from analysis. In addition, another steer from the NO-TAN and NO-IMP treatment did not use the self-feeder during the 90-d of the study; this individual was also removed from analysis. Data were analyzed as a completely randomized design with animal considered an experimental unit. The model diagnostics included testing for normal distribution of the error residuals and homogeneity of variance. These assumptions adequately held. Package ‘emmeans’ was used to calculate least square means of treatment-level responses for each period via the *emmeans*() function (Lenth, 2024). A 2-way interaction between implant status and tannin supplementation was not detected (*P* ≥ 0.05) for performance, emissions, or nutrient utilization analyses; therefore, this interaction is not discussed below. Treatment was included as a fixed effect in a linear mixed-effects model. Due to the change in CH_4_ and CO_2_ concentration sensors between the 2 periods (early = days 0 to 45; late = days 46 to 90), period was used as a random intercept to account for sensor variance across the whole 90-d study. The function *lmer*() within package lme4 was used to assess treatment outcomes. For analysis, individuals with ≥10 good (animals that remained in AHCS system for 3 to 8 min; Thompson et al., 2021) visits in each period were selected for final analysis (2 animals had less than 10 visits and were removed from analysis). Significance was determined at *P* ≤ 0.05, and tendency was determined at 0.05 < *P* ≤ 0.10. R software was used for all analyses (R Development Core Team, 2023, v. 4.1.2).

## Results

No differences were detected in the initial BW of the animals (Table 3), indicating an equal distribution of BW among treatments as planned. There was no significant interaction (*P* ≥ 0.05) between growth hormone implant and tannin supplement, indicating no additive effect of low-level tannin supplementation in this study. Therefore, the main effect of each technology is reported and data were analyzed with treatment levels as follows: NO-IMP (all animals that did not receive growth-hormone implant) vs. IMP (all animals that did receive growth-hormone implant), and NO-TAN (all animals that did not receive tannin supplement) vs. TAN (all animals that did receive tannin supplement). Additionally, measurements for the first and second 45 d of the study were analyzed separately as a replacement of an earlier AHCS (*n* = 1,304 spot samples) to a later model AHCS (*n* = 1,661 spot samples) resulting in an 18% decrease in CH_4_ (g/d) production; mean (± SE): 228.29 (1.72) vs. 186.03 (1.17).

**Table 3.** Effects of growth implant and tannin supplement on performance of stocker steers grazing pivot-irrigated pasture at ARDEC, Fort Collins, CO, USA

	Growth implant^1^		Tannin^2^			*P*-value		
Item	NO-IMP	IMP	NO-TAN	TAN	SEM	IMP	TAN	IMP × TAN
*n*, animals	8	10	9	9				
Initial BW, kg	344	344	345	343	2.88	0.89	0.56	0.39
Final BW, kg	426	430	430	426	4.50	0.56	0.44	0.09
ADG, kg/d^3^	0.97	1.01	1.02	0.96	0.09	0.57	0.26	0.41
Total DMI, kg/d^4^	9.07	9.18	9.19	9.05	0.36	0.17	0.06	0.75
Forage^5^	8.51	8.56	8.55	8.51	0.34	0.34	0.28	0.05
Supplement	0.34	0.33	0.36	0.31	0.04	0.99	0.43	0.43
Alfalfa pellet	0.23	0.28	0.28	0.23	0.02	0.03	0.05	0.24

^1^NO-IMP = no implant given; IMP = implanted with Revalor-G (Merck Animal Health; Rahway, NJ, USA); irrespective of tannin-supplementation.

^2^NO-TAN = no tannin supplement; TAN = tannin supplemented (targeted for 0.30% of DMI) with SilvaFeed BX (Silva Team, San Michele Mondovi CN, Italy); irrespective of implanting.

^3^Whole study period = days 0 to 90.

^4^Total DMI = estimated forage intake + sweetfeed mix intake + AHCS bait (alfalfa pellet) intake.

^5^Forage intake was estimated using NRC (2000) special considerations for all-forage diets intake equation.

### Animal Performance

There was no effect (*P* ≥ 0.44) of treatments on cattle FBW (Table 3). Implanted steers had greater alfalfa pellet intake compared to NO-IMP steers (*P* = 0.03). Moreover, non-tannin supplemented steers tended (*P* = 0.05) to have greater alfalfa pellet intake and tended (*P* = 0.06) to have greater total DMI compared to TAN steers, with no main effect (*P* ≥ 0.17) of treatments for any other growth performance variable. Estimated daily tannin intake was 27.5 g/hd/d (0.24% of DMI) for TAN steers.

### Gas Emissions

Neither growth implants (*P =* 0.15) nor tannin supplementation (*P =* 0.49) had an effect on CH_4_ production (g/hd/d) over the 90 d of the study (Table 4). Similarly, neither growth implants (*P* = 0.08) nor tannin supplementation (*P* = 0.22) had effects on methane yield (MY; g CH_4_/kg DMI). Growth implants had no effect on EI (*P* = 0.20), CO_2_ production (*P =* 0.90), O_2_ consumption (*P =* 0.80), or late-season H_2_ production (*P =* 0.98). The inclusion of tannin as a supplement had no effect on EI (*P* = 0.22), CO_2_ production (*P* = 0.90), O_2_ consumption (*P* = 0.57), or H_2_ production (*P* = 0.82). Table 5 shows that empirically measured CH_4_ emissions from the AHCS were 10.7% and 5.2% higher than the IPCC Tier 2 (2019) model and Thompson et al. (2019) model predictions, respectively.

**Table 4.** Effects of growth implants and tannin on emission measurements of stocker steers grazing pivot-irrigated pasture at ARDEC, Fort Collins, CO, USA

	Growth implant^1^		Tannin^2^				*P*-value		
Item	NO-IMP	IMP	NO-TAN		TAN	SEM	IMP	TAN	IMP × TAN
*n*, animals^3^	7	9	7	9					
Visits per animal	62.5	80.4	77.9		67.0	12.9	—	—	—
Visits per day	0.78	1.00	0.97		0.84	0.16			
CH_4_, g/d^4^	220	212	214		218	23.5	0.15	0.49	0.54
EI, g CH_4_/kg gain	235	218	219		234	42.0	0.20	0.22	0.27
MY, g CH_4_/kg total DMI	24.5	23.2	23.4		24.2	3.52	0.08	0.22	0.50
CO_2_, g/d	7650	7664	7676		7638	356	0.87	0.90	0.24
O_2_ consumption	5601	5625	5652		5573	318	0.80	0.57	0.41
H_2_ (g/d)^5^	1.02	1.02	1.00		1.03	0.10	0.98	0.82	0.37

^1^NO-IMP* =* no implant; IMP* =* implanted with Revalor-G (Merck Animal Health; Rahway, NJ, USA); irrespective of tannin-supplementation.

^2^NO-TAN = no tannin supplement; TAN = tannin supplemented (targeted for 0.30% of DMI) with SilvaFeed BX (Silva Team, San Michele Mondovi CN, Italy); irrespective of implanting.

^3^Only animals with ≥10 “good” (3 to 8 min in duration) visits were selected for emissions-related analysis within period.

^4^Whole study period = days 0 to 90.

^5^Only days 46 to 90 evaluated.

**Table 5.** Measured and estimated absolute CH_4_ emissions

Item	AHCS measured emissions	IPCC tier 2^1^	Thompson et al., (2019) ^2^	*P*-value
CH_4_, g/d	216^a^	194^b^	205^b^	<0.01

^1^ IPCC (2019). Chapter 10: emissions from livestock and manure management. Available at https://www.ipcc-nggip.iges.or.jp/public/2019rf/pdf/4_Volume4/19R_V4_Ch10_Livestock.pdf. Accessed February 1, 2024.

^2^Thompson et al. (2019)

### Blood Urea Nitrogen, Nitrogen, Creatinine, and Phosphorus

Neither growth implants nor the inclusion of tannin as a supplement affected urinary N (ppm N*; P =* 0.30 and *P =* 0.49, respectively), creatinine (mg/dL*; P =* 0.46 and *P =* 0.45, respectively), fecal N (% N; *P =* 0.98 and *P =* 0.74, respectively), or fecal phosphorus (%P_2_O_5_; *P =* 0.95 and *P =* 0.77, respectively; Table 6). Growth implants did not increase BUN (*P* = 0.08), while tannin supplementation also did not affect BUN (*P =* 0.12).

**Table 6.** Effect of tannin supplementation and growth implants on blood metabolites, creatinine, and N and P metabolism of stocker steers

	Growth implant^1^		Tannin^2^			*P*-value		
Item	NO-IMP	IMP	NO-TAN	TAN	SEM	IMP	TAN	IMP × TAN
*n*, animals	8	10	9	9				
Blood urea nitrogen, mg/dL^3^	12.1	13.0	12.3	13.0	0.48	0.08	0.12	0.79
Urinary N, ppmN	1063	1496	1445	1162	275	0.30	0.49	0.24
Creatinine, mg/dL	12.3	17.4	17.4	12.9	4.01	0.46	0.45	0.41
Fecal N, % N	1.96	1.94	1.94	1.96	0.05	0.98	0.74	0.55
Fecal P, % P_2_O_5_	1.09	1.09	1.08	1.10	0.07	0.95	0.77	0.65

^1^NO-IMP* =* no implant; IMP* =* implanted with Revalor-G (Merck Animal Health, Rahway, NJ, USA), irrespective of tannin supplementation.

^2^NO-TAN = no tannin supplement; TAN = tannin supplemented (targeted for 0.30% of DMI) with SilvaFeed BX (Silva Team, San Michele Mondovi CN, Italy); irrespective of implanting.

^3^Whole study period = days 0 to 90.

## Discussion

In this experiment, forage intake was similar between non-supplemented and tannin-supplemented steers and ranged from 8.34 to 8.92 kg DM/d for the 90-d study. All steers had the same opportunity to graze the same forage, therefore we assume the nutrient content of the forage they were consuming was similar. There are individual animal factors that can affect forage intake such as body composition, sex, age, physiological state, and frame size (NRC, 2000; NASEM, 2016). Although we did not measure these variables, all cattle were steers and were sourced from the same herd at the same ranch, the CSU John E. Rouse- Beef Improvement Center, Saratoga, WY, USA. Therefore, we can assume genetics and environment are similar between cattle. Large ruminants also search for preferred forage, such as new growth or green plant material, and this pattern continues until almost no green is left, prior to moving to lower quality more fibrous plant material (Lyons and Machen, 2012; Raynor et al., 2016) and this behavior can vary between individuals. This selective grazing pattern is a challenge to quantify accurately, and the current study used the quadrat method described by Thompson et al. (2021) to collect forage samples, which is a non-selective method that is not indicative of what cattle prefer. Additionally, there may be some variation in CP and ADF content in the forage of individual animal intake, which would alter the output of the NRC (2000) predicted forage intake equation used in the current study. Thus, the high variability in forage chemical composition observed across this 90-d study may have also reduced our ability to accurately predict DMI and infer responses of steer CH_4_ yield to treatments correctly.

The current study did not find low-level tannin supplementation to alter forage DMI. Comparable results to the current study were reported by Beauchemin et al. (2007) when quebracho tannin extract supplement was included in a basal diet that consisted of approximately 70% forage fed to beef steers and heifers at 0%, 1%, and 2% of dietary DM, yielding no effect on DMI. Aboagye et al. (2018) found similar results to the present study observing no effect on DMI, when a blend (50:50) of condensed quebracho tannin and hydrolysable chestnut tannin was fed at 1.5% dietary DM to steers fed a high forage diet made up of alfalfa silage and barley silage. However, Piñeiro-Vázquez et al. (2018) found a reduction in DMI at 4% tannin inclusion rates when compared to no inclusion in *Bos taurus* × *B. indicus* crossbred heifers fed a low-quality fresh chopped Taiwan grass (*Pennisetum purpureum*) diet supplemented with quebracho tannin extract at 0%, 1%, 2%, 3%, and 4% of DMI. These results suggest that tannin can be added at low (0.30% DM) or high (up to 3% DM) levels to a forage-based diet and not affect forage DMI; however, fed at levels of 4% DM or more may negatively affect DMI in high forage diets. However, conflicting results were reported by Norris et al. (2020) evaluating condensed tannin supplemented at 0%, 1.5%, 3%, and 4.5% DM to steers fed a 56.5% roughage diet and reported that supplementing quebracho condensed tannin at 4.5% DM increased DMI compared to steers that received no tannin supplement. Therefore, diet composition and quality may contribute to the variable results reported in the literature.

In the current study, total DMI includes forage, alfalfa pellet, and sweetfeed intake. The non-supplemented steers tended to visit the AHCS more frequently which resulted in an increased amount of alfalfa pellet bait feed intake. Therefore, because alfalfa pellet bait intake (from the AHCS) was greater in non-supplemented steers, the overall, or total DMI tended to be greater for steers not receiving the tannin supplement. Average NDF content in the current study was 54.3% DM which is higher than reported by Beauchemin et al. (2007) and Aboagye et al. (2018) at 45.1% and 43.8% DM, respectively. In a pasture with higher NDF content, the additional impact of tannins on fiber digestion may further reduce overall digestibility, which could reduce intake. Furthermore, it has been reported that condensed tannin or hydrolysable tannins at >50 g/kg of DM would result in a reduction in DMI. Moreover, several reviews have suggested that the reduced intake could be due to a reduction of palatability of diets when tannin is supplemented, decreased rate of digestion in the rumen, and the development of toxicity (Frutos et al., 2004; Patra and Saxena, 2011).

Estimated forage intake ranged from 8.39 to 8.93 kg/d for implanted steers and 8.34 to 8.77 kg/d for NO-IMP steers with similar intake for implanted and non-implanted steers for the 90-d study. Moreover, growth implants appeared to positively affect total DMI (estimated forage plus alfalfa pellet and sweetfeed; 9.07 vs. 9.18 kg DM/d for non-implanted and implanted, respectively), although the outcome was not statistically different. These results are similar to those reported by Wileman et al. (2009), Rumsey et al. (1999), Parr et al. (2011a), and Song and Choi (2001). Wileman et al. (2009) conducted a meta-analysis of conventional versus nonconventional beef production and concluded that implanted steers increased DMI by 0.53 kg/d relative to non-implanted steers. Similar results were observed by Parr et al. (2011b) when crossbred cattle on a finishing diet were implanted with Revalor-S (120 mg of trenbolone acetate [TBA] and 24 mg of estradiol), Revalor-IS followed by Revalor-S (reimplanted at 68 to 74 d), or Revalor-XS (200 mg of TBA and 40 mg of estradiol) (Merck Animal Health, Rahway, NJ, USA), yielding an increase in DMI in all growth implant treatment groups compared to the control group. Growth implants stimulate DMI (Smith and Johnson., 2020); therefore, the finding of implants to numerically increase total DMI compared to the non-implanted animals is expected.

### Animal Performance

Previous research has reported that tannins have varying effects on cattle growth performance. For example, Ebert et al. (2017) reported similar results to the current study when supplementing condensed tannin manufactured by Silvafeed at 0%, 0.5%, and 1% DM to beef cattle, yielding no difference in ADG. Additionally, there was no difference in FBW between tannin-supplemented and non-supplemented steers, which adds to findings presented throughout the literature that tannin supplementation has mixed outcomes for beef cattle growth performance.

The addition of a growth implant did not significantly affect FBW or ADG over the full 90-d of the study, which conflicts with other studies evaluating implant response in grazing beef cattle. McMurphy et al. (2011) implanted steers grazing summer pasture with Ralgro (36 mg of zeranol) or Component TE-G with Tylan (40 mg of TBA and 8 mg of estradiol; 29 mg of tylosin tartrate) and reported an increase in FBW and an 8.1% improvement in ADG during the first 95 d regardless of implant type employed in the trial. Component TE-G contains the same amount of active ingredients as Revalor-G used in the current study; however, the product evaluated here lacked inclusion of Tylan. Additionally, Beck et al. (2014) implanted steers grazing wheat pastures in the fall months with Component TE-G (40 mg of TBA and 8 mg of estradiol) and reported that the addition of the growth implant increased ADG by 0.14 kg/d. The addition of growth implants can increase ADG by 6% to 16% in grazing cattle compared to control animals (Shockey et al., 1996; Kuhl and Blasi, 1998; McMurphy et al., 2011; Parr et al., 2011a, b; Beck et al., 2014; Tibbitts et al., 2017). Additionally, the lack of effect of growth implant on ADG presented in the current study is contrary to previous studies specifically evaluating Revalor-G in grazing beef cattle, where a 6% to 8.5% increase in ADG has been observed (Shockey et al., 1996; Kuhl and Blasi 1998; Tibbitts et al., 2017). While the current study lacked a statistical difference in ADG between implanted cattle and non-implanted cattle, there was a slight 4.1% increase in ADG in implanted cattle. Beck et al. (2014), using a similar implant (Component TE-G, 40 mg trenbolone acetate and 8 mg estradiol) on grazing steers, reported implanted steers had an 11% increase in ADG compared to the control. Similarly, McMurphy et al. (2011), also investigating Component TE-G, found an 8% ADG increase in steers grazing summer pasture. In the current study, the implanted steers grazing summer pasture had a numerically greater ADG (1.01 vs. 0.97 kg/d) compared to the no implant-control for the full 90 d of the study. This outcome could be due to an initial implant response leveling off or to varied forage quality in the latter half of the study.

### Gaseous Emissions

The mean daily CH_4_ emissions for all animals observed in this experiment was 216 g/d (Table 5), which was compared to the IPCC Tier 2 model (IPCC, 2019) and the Thompson et al. (2019) prediction equations. Our measured CH_4_ emissions from the AHCS were 10.7% and 5.2% higher than the IPCC Tier 2 (2019) model and Thompson et al. (2019) model predictions, respectively. However, both predicted CH_4_ equations use DMI as a parameter; thus, using an estimated DMI may explain the variation in CH_4_ emission outcomes. Generally, measured emissions from the current study, which accounts for variation in emissions sensors, are reasonable when compared to the literature. For instance, our measured emissions are 7% higher than that reported in Beck et al. (2018), where steers grazed native warm-season pasture and were supplemented with whole cottonseed or soybean and weighed 269 kg. Additionally, CH_4_ emissions measurements in this study were 20% higher than reported by Thompson et al. (2019), where steers and heifers weighed 262 and 240 kg, respectively, and grazed wheat forage.

Tannin supplemented at 0.24% DM did not alter CH_4_ emissions of grazing Angus steers. These results agree with those found by Beauchemin et al. (2007), who fed tannin supplement at up to 2% of DM to Angus heifers fed a forage-based diet (70%) and reported no effect on CH_4_ emissions. Although Beauchemin et al. (2007) found no effect of quebracho tannin extract supplement on CH_4_ production, protein binding was evident because there was less ruminal NH_3_ concentration. Conversely, Carulla et al. (2005) fed *Acacia mearnsii* tannin (condensed tannin) at 2.5% DM to 6 growing castrated male lambs fed 3 different basal haylage diets. Although Carulla et al. (2005) reported no interaction between basal diet and the addition of tannin supplement, they did find a 12% reduction in enteric CH_4_ production when lambs were supplemented with 2.5% condensed tannin. The current study used a mixture of quebracho tannin (condensed) and chestnut tannin (hydrolysable). Tannins from different plants vary in their ability to bind to carbohydrates and proteins (McAllister et al., 2005). Therefore, it is possible that tannin derived from quebracho tree bark is less effective at reducing CH_4_ production compared to other tannin sources, such as *A. mearnsii* tannin (Carulla et al., 2005).

To further support this theory, Aboagye et al. (2018) found no effect of feeding a mixture of chestnut tannin (hydrolysable) and quebracho tannin (condensed) at 1.5% DM on CH_4_ production. Other studies have indicated feeding condensed tannin-containing forages to ruminants reduces CH_4_ emissions (Waghorn et al., 2002; Pinares-Patiño et al., 2003; Carulla et al., 2005; Puchala et al., 2005). The tannin-containing forages in these studies varied, and the percent tannin included in the diet varied. However in most of these studies, there were changes in forage quality, such as lower NDF, which could be associated with a reduction in CH_4_. For example, Puchala et al. (2005) grazed angora goats (*Capra hircus*) on *Sericea lespedeza* containing 17.7% (DM) condensed tannin, and crabgrass/tall fescue containing 0.5% (DM) condensed tannin, where they reported CH_4_ emissions were 30% lower for goats grazing *Sericea lespedeza* than goats grazing crabgrass/tall fescue. However, the NDF content (% of DM) of *S. lespedeza* was 28% lower than crabgrass/tall fescue. Therefore, because lower-fiber diets are associated with lower CH_4_ emissions (Johnson and Johnson, 1995), a reduction in absolute CH_4_ production could be due to the change in nutrient composition. In the current study, the NDF value of the forage was, on average, 54.4% DM which is greater than the 45.1% DM reported by Beauchemin et al. (2007) when feeding heifers a 70% forage diet. In addition to the lower than expected intake of tannin supplement, the reduction of NDF in the forage could partially explain the lack of effect of tannin supplementation on CH_4_ production. Fiber tends to be less digestible due to added structural carbohydrates (cellulose, hemicellulose, and lignin), which can be more resistant to enzymatic breakdown in the digestive system and require microbial fermentation in the rumen (Varga and Kolver, 1997). Other studies utilize a higher dose of tannin supplement compared to our current study. For example, Piñeiro-Vazquez et al. (2018) observed the greatest reductions in CH_4_ production at 3% and 4% tannin inclusion rates compared to 0% and 1% tannin inclusion rates in crossbred heifers supplemented with quebracho tannin extract at 0%, 1%, 2%, 3%, and 4% of DM. However, Pineiro-Vazquez et al. (2018) found a reduction in DMI at the 4% inclusion rate which correlates to a reduction in CH_4_ production. The absence of an impact from the supplementation of blended chestnut and quebracho tannins on CH_4_ production reported here might be attributed to the dose employed (e.g., 0.24% DM vs. >2.0% DM), the type of tannin that was used, and the amount of dietary fiber. Additionally, tannin-supplemented animals did not consistently receive the complete dose of tannin supplement daily, which may be due to supplement intake being voluntary through the Smartfeed Pro self-feeder. McClain et al. (2020) further corroborate this outcome which offered supplements through the Smartfeed Pro to yearling heifers grazing dryland pastures. They found supplement intake appeared to be influenced by timing of herd section moves, which seemed to be related to forage quantity/quality of each pasture.

Enteric CH_4_ production in grazing environments can be directly related to animal growth due to the increase in DMI, which in turn, increases ruminal fermentation and methanogenesis (Gerber et al., 2013; Thompson and Rowntree, 2020; Min et al., 2022). In the present study, implanted cattle compared to non-implanted cattle for the 90 d study showed no difference in FBW or whole study ADG but there was a numerical increase in estimated forage intake. Absolute CH_4_ production ranged from 208 to 289 g CH_4_/d for all cattle; however, the addition of growth implants did not affect CH_4_ production. These results conflict with those reported by Stackhouse et al. (2012), who conducted a partial lifecycle assessment using the Integrated Farm System Model which suggested that implanted Angus cattle emit 11% higher CH_4_ (kg CO_2_e/animal) than the Angus control in the stocker segment alone. This result may be due to growth-implanted cattle having increased DMI. Thus, there is still considerable uncertainty about implantation impact on enteric CH_4_ production in grazing steers.

Although previous literature shows that increasing DMI and growth performance influences CH_4_ production (Min et al., 2022), intake and concomitant performance also decrease EI based on g CH_4_ emitted per kg of gain (Stackhouse et al., 2012). Therefore, the slight 4% increase in ADG in the implanted cattle and no difference in CH_4_ production may explain the observed decrease in EI by 7% in implanted cattle compared to non-implanted cattle.Increasing animal performance is proposed as one of the more successful mitigation strategies to decrease GHG emissions from cattle production per unit of product produced. By improving growth, beef cattle meet their market-ready endpoint on fewer days on feed than cattle grown without growth-promoting technologies (Stackhouse et al., 2012). Furthermore, Basarab et al. (2012) considered emissions throughout the beef production cycle, where they reported harvest of non-implanted cattle at the same BW as implanted cattle resulted in an increase of 12 to 17 days on feed, and the longer feeding duration resulted in a 10.5% to 15.8% increase in the carbon footprint. Although the current study did not measure beyond the backgrounding stage of production, a slight increase in ADG shows cattle implanted with growth-promoting technologies have the opportunity to reach their harvest endpoint on fewer days on pasture.

### Blood Urea Nitrogen, Nitrogen, Creatinine, and Phosphorus

In the current study, the inclusion of a growth implant had no effect on urinary N, creatinine, fecal N, or fecal P. A tendency for implanted steers to have increased BUN compared to the control animals for the 90-d experiment suggests that implants may affect N excretion. Differing results from the current study were reported when Bryant et al. (2010) implanted heifers with 200 mg of TBA, 20 mg of estradiol, and supplemented 250 mg of ractopamine—yielding a decreased serum urea nitrogen (SUN) compared to the control. The mixed outcomes in the literature could be due to the variation in active ingredients of the Revalor product and sampling methods. Future research should include how active ingredients in growth implants impact BUN concentrations.

Lavery and Ferris (2021) explain that the timing of sampling can influence BUN concentrations as BUN levels fluctuate throughout the day, with the highest levels normally detected 4 to 6 h after feeding. The current study collected blood samples after a 12-h fast and found implanted cattle tended to have increased BUN concentration. Samples for BUN analysis may have been collected in this experiment at a time when BUN concentration was low due to no recent feed intake. This was done in the current study due to practicality and caution over reducing performance from gathering and weighing too frequently. However, future grazing studies should take this methodological problem into consideration by collecting multiple samples throughout the day to estimate N excretion.

Tannins have the affinity to bind to protein in the rumen, increasing bypass protein into the small intestine and decreasing ruminal protein degradation; therefore, it can be assumed that tannins in the diet could decrease the amount of N excreted in the urine and increase the amount of N excreted in the feces (Carulla et al., 2005; Aboagye et al., 2018). In the present study, the inclusion of tannin at 0.24% DM had no effect on BUN, urinary N, creatinine, or fecal N. Stewart et al. (2019) fed condensed tannin-containing legumes: bird’s-foot trefoil (*Lotus corniculatus*) and sainfoin (*Onobrychis* sp.), and a hydrolysable tannin-containing forb: small burnet (*Sanguisorba minor*) at 2.5%, 0.6%, and 4.5% DM, respectively, to beef cows and heifers, where they reported all tannin-containing diets reduced BUN compared to control animals. Further, evidence has shown that feeding tannin levels as low as 0.25% DM can decrease BUN levels; therefore, the lack of effect found in the present study may be attributed to the timing of blood sample collection.

## Conclusion

Growth implants slightly increased total DMI and ADG in this study although results were non-significant. Revalor-G did not affect N utilization or CH_4_ production; however, it numerically decreased EI and tended to decrease MY during the 90-d experiment. The inclusion of growth implants in grazing stocker steers shows promise in increasing ADG while decreasing EI in a non-confined setting, yet more work is needed to directly examine the effect of growth implants on DMI and CH_4_ emissions in a grazing environment.

The low-level inclusion of tannin in the diet did not affect growth performance or N utilization; however, tannin decreased the intake of alfalfa pellets (from the AHCS) and, in turn, tended to reduce total DMI. Tannin supplementation also did not reduce absolute CH_4_ emissions. The lack of effect of tannin inclusion on CH_4_ production may have resulted from steers not consuming the full dose of tannin daily due to variable individual intake. The type of tannin used and the level of tannin supplementation is varied among published studies; thus, the results from tannin supplementation to reduce CH_4_ emissions are variable. Investigation is needed to determine the most beneficial type of tannin to be used and the range at which tannins can be supplemented to mitigate emissions from grazing stocker steers on improved pasture. Investigators should consider the difficulty of supplementing grazing cattle on pasture and account for the variation of daily intake on an individual animal basis.

Further investigation is required to determine how different growth implant active ingredients and dosage of tannin might be used to alter absolute CH_4_ production, EI, and MY in a grazing environment. Investigators should consider the difficulty of accurately determining DMI in a grazing environment and how variation in DMI impacts reporting MY. Such contributions can bolster our understanding of the efficacy of stacking multiple technologies for improving animal performance while simultaneously reducing GHG emissions.
